# Consumo alimentar da população urbana em um município da Amazônia Legal, nos eventos climáticos de inundação e seca: estudo comparativo

**DOI:** 10.1590/0102-311XPT110223

**Published:** 2024-10-11

**Authors:** Patrícia dos Santos Guimarães, Gleiciane da Silva Costa, Amanda Forster Lopes, Michel Nasser Corrêa Lima Chamy, Vera Lucia Conceição de Gouveia Santos

**Affiliations:** 1 Instituto de Saúde e Biotecnologia, Universidade Federal do Amazonas, Coari, Brasil.; 2 Escola de Enfermagem, Universidade de São Paulo, São Paulo, Brasil.

**Keywords:** Alimentação Regional, Alterações Climáticas, Inundações, Secas, Regional Food, Climate Changes, Floods, Droughts, Comida Regional, Alteraciones Climáticas, Inundaciones, Sequías

## Abstract

O objetivo foi comparar o consumo alimentar da população de Coari, Amazonas, Brasil, segundo a Classificação NOVA, durante as fases hidrológicas de seca e inundação dos rios amazônicos. Realizou-se um estudo epidemiológico, de base populacional e transversal. A amostra foi composta por 457 indivíduos adultos e a coleta de dados foi realizada em dois momentos, mediante um instrumento sociodemográfico, um recordatório alimentar de 24 horas, e um questionário de frequência alimentar adaptado para os hábitos locais. Os dados foram analisados pelo programa estatístico R versão 4.2.4, por meio dos testes qui-quadrado de Pearson, exato de Fischer e de Bhapkar. A amostra foi composta predominantemente pelo sexo feminino (seca = 70%/inundação = 71,2%) e pardos (seca = 65,4%/inundação = 66,2%). As refeições (café da manhã, almoço e jantar) foram realizadas pela maior parte dos entrevistados. O lanche da tarde foi a refeição intermediária mais realizada, principalmente na inundação (274/70,2%). Predominou-se o consumo de alimentos *in natura* ou minimamente processados nas três principais refeições (95%). Os ultraprocessados são pouco ou não são consumidos e foram citados especialmente na seca (152/33,3%; p = 0,007). Em contrapartida, o consumo de alimentos regionais (tucumã, beiju, farinha de tapioca e açaí) aumentou durante a inundação (p < 0,001). O consumo de alimentos *in natura* ou minimamente processados continua sendo a base da alimentação no interior do Amazonas, predominando alimentos regionais na inundação e alimentos processados e ultraprocessados na seca, demonstrando a influência, ainda que sutil, das fases hidrológicas no consumo alimentar dessa população.

## Abstract (en)

The aim of this study was to compare the food consumption of the population of Coari, Amazonas State, Brazil, according to the NOVA Classification, during the hydrological phases of drought and flooding of the Amazon rivers. An epidemiological, population-based, cross-sectional study was carried out. The sample consisted of 457 adult individuals. Data were collected in two stages using a sociodemographic instrument, a 24-hour food recall and a food frequency questionnaire adapted to local habits. The data were analyzed using the statistical program R version 4.2.4, using Pearson’s chi-square, Fischer’s exact and Bhapkar’s tests. The sample was predominantly female (drought = 70%/flood = 71.2%) and brown (drought = 65.4%/flood = 66.2%). Most of the interviewees ate meals (breakfast, lunch and dinner). Afternoon snacks were the most common intermediate meal, especially during flooding (274/70.2%). In natura or minimally processed foods predominated at the three main meals (95%). Ultra-processed foods were consumed little or not at all and were mentioned especially during the drought (152/33.3%; p = 0.007). On the other hand, consumption of regional foods (*tucumã*, *beiju*, *tapioca* flour and *açaí*) increased during the flood (p < 0.001). Consumption of in natura or minimally processed foods continues to be the mainstay of the diet in the interior of Amazonas, with a predominance of regional foods during the flood and processed and ultra-processed foods during the drought, demonstrating the influence, albeit subtle, of the hydrological phases on the food consumption of this population.

## Resumen

El objetivo de este estudio fue comparar el consumo de alimentos de la población de Coari, Amazonas, Brasil, según la Clasificación NOVA, durante las fases hidrológicas de sequía e inundación de los ríos amazónicos. Se trató de un estudio epidemiológico, poblacional y transversal. La muestra estuvo conformada por 457 individuos adultos. Los datos se recogieron en dos etapas mediante un instrumento sociodemográfico, un recordatorio de alimentos las 24 horas, y un cuestionario de frecuencia alimentar adaptado a las costumbres locales. Para el análisis de datos se utilizó el programa estadístico R versión 4.2.4, mediante las pruebas chi-cuadrado de Pearson, exacto de Fisher y de Bhapkar. Hubo un mayor predominio del sexo femenino (sequía = 70%/inundación = 71,2%) y de pardos (sequía = 65,4%/inundación = 66,2%). La mayoría de los entrevistados tuvieron sus comidas (desayuno, almuerzo y cena) en el período analizado. La merienda fue la comida intermedia más frecuente, especialmente en la inundación (274/70,2%). El consumo de alimentos *in natura* o mínimamente procesados predominó en las tres comidas principales (95%). Los productos ultraprocesados fueron poco o nada consumidos y se los mencionaron especialmente en la sequía (152/33,3%; p = 0,007). Por otro lado, el consumo de alimentos regionales (*tucumã*, *beiju*, harina de *tapioca* y *açaí*) tuvo un aumento durante la inundación (p < 0,001). El consumo de alimentos *in natura* o mínimamente procesados sigue siendo la base alimentar en el interior de la Amazonía, con predominio en el consumo de alimentos regionales en la inundación y de alimentos procesados y ultraprocesados en la sequía, lo que evidencia la influencia, aunque discreta, de las fases hidrológicas en el consumo de alimentos de esta población.

## Introdução

O consumo de alimentos ultraprocessados tem aumentado significativamente nas últimas décadas, gerando preocupações sobre potenciais efeitos adversos à saúde ^1^. Nesse atual cenário, acumula-se o corpo de evidências que indica a associação entre o consumo desses alimentos com obesidade e outras doenças crônicas não transmissíveis, tornando-se imprescindível compreender seus impactos potenciais sobre desfechos em saúde ^2^ ^,^ ^3^.

Uma alimentação adequada e saudável envolve, para além do biológico, aspectos ambientais, culturais, sociais, demográficos e econômicos, contexto fundamental a ser considerado em nível populacional para a adequada manutenção da saúde e prevenção das doenças crônicas não transmissíveis ^3^ ^,^ ^4^. Adotando esse conceito, o *Guia Alimentar para a População Brasileira* possui um olhar abrangente sobre a alimentação e aborda orientações embasadas no grau de processamento dos alimentos ^4^ ^,^ ^5^.

A Classificação NOVA, adotada pelo *Guia Alimentar para a População Brasileira*, agrupa os alimentos em quatro grandes grupos. Os *in natura* ou minimamente processados (grupo 1) são obtidos diretamente de plantas/animais ou sofreram alteração física sem adição de qualquer substância/ingrediente. Os ingredientes culinários processados (grupo 2) são os obtidos dos alimentos *in natura*, como óleos e gorduras, sal e açúcar, comumente utilizados para preparações culinárias. Os alimentos processados (grupo 3) possuem adição de sal, açúcar ou outro ingrediente culinário aos alimentos *in natura* ou minimamente processados. Por fim, os ultraprocessados (grupo 4) são formulações industriais cujos ingredientes consistem em substâncias alimentares de uso culinário inexistente ou raro cuja função é tornar o produto final altamente palatável ^4^.

Seguindo a tendência mundial, já se observam mudanças nos padrões de consumo de populações na Amazônia Ocidental em um processo de substituição de produtos locais, obtidos por meio da pesca, caça e agricultura de subsistência, por alimentos processados e ultraprocessados, obtidos em supermercados e outros estabelecimentos de comercialização de alimentos ^6^.

A Região Amazônica tem períodos bem distintos de secas e inundações que alteram o cotidiano dos indivíduos, afetando a disponibilidade de recursos e também a variedade de alimentos ^7^. No geral, o período da seca representa fartura, com possibilidade de cultivo e pesca, e na inundação, há escassez de produtos e aumento da necessidade da importação de alimentos para garantir o abastecimento local, onerando os custos e dificultando o acesso pela população de mais baixa renda ^8^.

Ainda que a Região Norte do Brasil apresente as menores médias de participação dos alimentos ultraprocessados no total de energia consumida ^9^, assim como a Nordeste, sabe-se que esses alimentos são associados a maior facilidade de preparo, praticidade na ingestão, baixo custo e fácil armazenamento ^10^ ^,^ ^11^, características que podem contribuir para o seu consumo em situações de altos preços e precariedade de acesso aos alimentos, conforme as oscilações nos níveis dos rios.

Tendo em vista que a saúde pode ser influenciada pela alimentação e, na Região Amazônica, essa pode estar relacionada aos eventos climáticos de inundação e seca dos rios locais, este estudo objetivou descrever e comparar o consumo alimentar da população urbana de Coari, Amazonas, durante as fases hidrológicas dos rios amazônicos segundo a Classificação NOVA.

## Material e métodos

Trata-se de uma análise secundária dos dados de um estudo epidemiológico de base populacional, do tipo transversal, intitulado *Padrão Intestinal da População Adulta Urbana e Eventos Climáticos em um Município do Interior da Amazônia Brasileira*, desenvolvido com adultos residentes na área urbana no Município de Coari, localizado no Médio Solimões, Amazonas.

A amostra do estudo foi constituída por todos os indivíduos adultos residentes na área urbana do Município de Coari ^12^. Por ser um estudo de análise secundária, a prevalência utilizada para o cálculo amostral foi a mesma do estudo original, sendo considerada a máxima esperada do desfecho de 50%, nível de 95% de confiança e margem de erro de 5% ^13^. Essa prevalência é adotada quando não há informação sobre o objeto de estudo na região.

A amostra representativa foi calculada em função do número de domicílios particulares permanentes do Município de Coari (10.382 residências distribuídas em 11 bairros), e após a correção pelo efeito do desenho *deff* igual a dois e acréscimo de 20% para taxa de não resposta, chegou-se a uma amostra de 445 domicílios ^12^ selecionados por meio de técnicas de processos probabilísticos com estratificação (representatividade em porcentagem da quantidade de domicílios sobre o total), visando à produção de resultados válidos para o conjunto do qual a amostra foi extraída. Para essa seleção, adotou-se a amostragem aleatória simples. Caso não fossem encontrados moradores nos domicílios sorteados, a equipe de coletores foi instruída a retornar até duas vezes e, não havendo sucesso, ele foi substituído por outro no mesmo bairro mediante novo sorteio. Para a definição de domicílio, considerou-se o local utilizado como habitação de uma ou mais pessoas.

Os critérios de inclusão dos residentes entrevistados foram: ter idade igual ou superior a 18 anos; aceitar participar do estudo nas fases hidrológicas investigadas (seca e inundação) e ser residente no município há mais de seis meses (para justificar o consumo alimentar ocorrido nas fases investigadas). Unidades não domiciliares, constituídas por estabelecimentos comerciais e coletivos como hospitais, clínicas, escolas, igrejas, clubes, quartéis e similares, foram excluídas. Todos os indivíduos presentes nos domicílios no momento da coleta que atendessem aos critérios de elegibilidade do estudo foram convidados a participar. Ao final, foram entrevistados 457 sujeitos na fase hidrológica da seca, e desses, 376 na inundação.

Portanto, a coleta de dados ocorreu em dois momentos: seca (novembro/dezembro de 2021) e inundação (maio/junho de 2022). Foi realizada por uma equipe composta de alunos regulamente matriculados nos cursos de Enfermagem e Nutrição do Instituto de Saúde e Biotecnologia da Universidade Federal do Amazonas (UFAM), devidamente capacitados e supervisionados pela pesquisadora principal do projeto. Para a coleta de dados, utilizou-se um instrumento subdividido em sessões: questionário sociodemográfico para caracterização da amostra; recordatório alimentar de 24 horas (R24h) ^14^; e tabela de frequência de consumo alimentar (TFCA).

As variáveis sociodemográficas de interesse investigadas neste estudo foram sexo, idade, raça, religião, escolaridade e estado civil. As variáveis sobre alimentação foram a frequência das refeições referidas pela amostra do estudo: café da manhã; lanche da manhã; almoço; lanche da tarde; jantar; e ceia.

O R24h foi conduzido conforme o *Multiple-Pass Method* (MPM; Método de Passagens Múltiplas), que corresponde a uma técnica de abordagem que tem como objetivo estimular uma resposta e aumentar a precisão da informação ^15^. A TFCA foi utilizada em estudo prévio para caracterização do consumo alimentar da Região Amazônica ^16^ e adaptada para este estudo, sendo composta por uma lista de 13 alimentos de consumo comum e 12 alimentos considerados regionais habitualmente consumidos pela população amazonense. Para identificar diferenças de consumo entre os dois momentos hidrológicos analisados, realizou-se a comparação entre frequência de consumo dos alimentos referidos a partir da TFCA.

Os alimentos informados nas refeições foram categorizados de acordo com a Classificação NOVA de alimentos ^4^. Para fins deste estudo, foram considerados os quatro grupos da Classificação NOVA segundo o grau de processamento. Contudo, os alimentos do grupos 2 e 3 foram aglutinados em uma única categoria, ficando da seguinte forma: alimentos *in natura* ou minimamente processados (grupo 1); ingredientes culinários processados e alimentos processados (grupo 2+3); e alimentos ultraprocessados (grupo 4).

Os dados provenientes de ambos os instrumentos foram analisados utilizando o programa R, versão 4.2.4 (http://www.r-project.org), por meio de estatística descritiva e com frequências absolutas e relativas. Os testes utilizados para verificar a associação das variáveis de resposta categórica à alimentação (R24h e TFCA) foram o de Bhapkar, qui-quadrado (χ^2^) de Pearson e o exato de Fisher. O teste de Bhapkar é considerado uma extensão do teste de McNemar, que é bem mais comum, e neste estudo foi utilizado para comparar as variáveis categóricas entre os dois períodos quando o mesmo sujeito respondeu nas duas situações (seca e inundação). Nas poucas variáveis que temos respostas de sujeitos diferentes nos dois períodos, temos a descaracterização de medidas pareadas e foi usado os testes qui-quadrado de Pearson ou exato de Fisher.

Para todas as análises estatísticas do estudo, considerou-se a significância ao nível de 5% (p < 0,05).

O projeto de pesquisa foi aprovado pelo Comitê de Ética e Pesquisa da UFAM (processo nº 102559/2021 e CAAE 51494521.2.0000.5020). Todos os participantes foram esclarecidos acerca dos objetivos da pesquisa, manifestando seu consentimento em participar por meio da assinatura do Termo de Consentimento Livre e Esclarecido (TCLE).

## Resultados

As características sociodemográficas dos participantes do estudo, em ambas as fases hidrológicas, são apresentados na Tabela 1.

**Tabela 1:** Características sociodemográficas da amostra do estudo, segundo as fases hidrológicas de seca e inundação. Coari, Amazonas, Brasil, 2022.

Características	Seca (n = 457)		Inundação (n = 376)	
	n	%	n	%
Sexo				
Feminino	320	70,0	268	71,2
Masculino	137	30,0	108	28,8
Idade * (anos)				
18-29	140	30,6	102	27,1
30-39	82	17,9	70	18,6
40-49	80	17,5	68	18,1
50-59	61	13,4	55	14,6
≥ 60	94	20,6	81	21,6
Cor/Raça				
Branco	51	11,2	41	10,9
Mulato	72	15,8	59	15,7
Negro	35	7,6	27	7,2
Pardo	299	65,4	249	66,2
Religião				
Ateu	3	0,7	2	0,5
Católico	255	55,8	211	56,1
Espírita	2	0,4	2	0,6
Evangélico	180	39,4	149	39,6
Outro	17	3,7	12	3,2
Escolaridade				
Analfabeto	36	7,9	31	8,2
Ensino Fundamental incompleto	101	22,1	84	22,4
Ensino Fundamental completo	29	6,3	28	7,5
Ensino Médio incompleto	26	5,7	14	3,7
Ensino Médio completo	171	37,4	137	36,4
Ensino Superior incompleto	36	7,9	33	8,8
Ensino Superior completo	58	12,7	49	13,0
Estado civil				
Solteiro	180	39,4	143	38,0
Casado	114	24,9	99	26,4
União estável	111	24,3	90	23,9
Separado/Divorciado	16	3,5	15	4,0
Viúvo	36	7,9	29	7,7

* Seca: média = 42,9 anos; desvio padrão = 18,1; mediana = 40; inundação: média = 44,0 anos; desvio padrão = 17,8; mediana = 42.

Observa-se que a amostra do estudo foi composta principalmente por mulheres (320/70% na seca e 268/71,2% na inundação) e indivíduos que se autodeclaram pardos (seca = 299/65,4%; inundação = 249/66,2%), e cerca de um quinto da amostra tinha Ensino Superior, completo ou não. A idade média, o desvio padrão (DP) e a mediana dos residentes foram bastante similares em ambas as fases hidrológicas.

Na Tabela 2 são apresentadas a frequência de consumo das refeições e da presença de alimentos segundo o grau de processamento pela Classificação NOVA.

**Tabela 2:** Frequência de consumo das refeições e da presença de alimentos segundo o grau de processamentos pela Classificação NOVA conforme a fase hidrológica do rio, seca e inundação. Coari, Amazonas, Brasil, 2022.

Tipo de refeição e grupo de alimentos *	Seca (n = 457)		Inundação (n = 376)		Valor de p
	n	%	n	%	
Café da manhã	434	95,0	355	94,4	0,612 **
Grupo 1	416	95,8	340	95,8	0,491 **
Grupo 2+3	310	71,4	217	61,1	0,001 **
Grupo 4	89	20,5	90	25,4	0,150 **
Lanche da manhã	72	15,7	51	13,6	0,263 **
Grupo 1	64	88,9	41	80,4	0,560 **
Grupo 2+3	14	19,4	11	21,6	1,000 **
Grupo 4	9	12,5	12	23,5	0,302 **
Almoço	446	97,6	370	98,4	0,763 **
Grupo 1	439	98,4	367	99,2	0,256 **
Grupo 2+3	10	2,2	3	0,8	0,051 **
Grupo 4	60	13,4	42	11,3	0,467 **
Lanche da tarde	275	60,2	264	70,2	0,017 **
Grupo 1	213	77,5	206	78,0	0,163 **
Grupo 2+3	98	35,6	71	26,9	0,195 **
Grupo 4	125	45,4	126	47,7	0,243 **
Jantar	402	88,0	329	87,5	0,799 **
Grupo 1	367	91,3	304	92,4	0,866 **
Grupo 2+3	16	4,0	10	3,0	0,796 **
Grupo 4	113	28,1	70	21,3	0,143 **
Ceia	26	5,7	25	6,6	0,773 **
Grupo 1	23	88,5	19	76,0	0,248 ***
Grupo 2+3	2	7,7	1	4,0	0,579 ***
Grupo 4	7	26,9	10	40,0	0,327 **

* Grupo 1: alimentos *in natura* ou minimamente processados; grupo 2+3: ingredientes culinários processados e alimentos processados; grupo 4: alimentos ultraprocessados;

** Teste de Bhapkar;

*** Teste qui-quadrado (χ^2^) de Pearson.

A Tabela 2 mostra que há predominância do consumo das principais refeições (café da manhã, almoço e jantar), sem diferenças entre os dois períodos analisados. Em relação às refeições intermediárias, o lanche da tarde foi realizado pela maioria dos entrevistados, com diferença estatisticamente significante (p = 0,017) entre as duas fases hidrológicas, seca (60,2%) e inundação (70,2%). A ceia foi a refeição realizada com menor frequência (cerca de 6% em ambas as fases).

Quanto ao consumo de alimentos segundo o grau de processamento, destaca-se o predomínio de alimentos do grupo 1 nas três principais refeições (> 95%). O consumo de alimentos ultraprocessados (grupo 4) oscilou entre 20% a 28% no café da manhã e no jantar, com menor participação no almoço (entre 11% e 13%). Houve diferença estatisticamente significante (p = 0,001) para o consumo dos alimentos do grupo 2+3 no café da manhã, menos frequente (61,1%) no período da inundação quando comparado ao período da seca (71,4%).

A Tabela 3 mostra baixo consumo de feijões em geral e consumo mais frequente das fontes proteicas como peixe de água doce, ovo e frango, comparativamente a carne bovina e suína. Nota-se que a prevalência de consumo frequente de legumes (88%) e verduras (64,8%) no período de seca foi superior à observada no período de inundação (80,3% e 59%, p = 0,005 e p = 0,006, respectivamente). Por outro lado, os ultraprocessados e alimentos tipo embutidos são, no geral, consumidos com baixa frequência ou não são consumidos por mais de dois terços da população. Para os ultraprocessados, observou-se prevalência de consumo frequente (33,3%) mais alta no período da seca que no de inundação (23,7%, p = 0,007).

**Tabela 3:** Frequência de consumo de alimentos nas fases hidrológicas de seca e inundação. Coari, Amazonas, Brasil, 2022.

Variável	Seca (n = 457)		Inundação (n = 376)		Valor de p *
	n	%	n	%	
Feijões					< 0,001
Nunca ou raramente	129	28,2	80	21,3	
Pouco frequente	227	49,7	167	44,4	
Frequente	101	22,1	129	34,3	
Tubérculos e raízes					< 0,001
Nunca ou raramente	269	58,9	213	56,7	
Pouco frequente	139	30,4	80	21,3	
Frequente	49	10,7	83	22,1	
Legumes					0,005
Nunca ou raramente	20	4,4	26	6,9	
Pouco frequente	35	7,7	48	12,8	
Frequente	402	88,0	302	80,3	
Verduras					0,003
Nunca ou raramente	88	19,3	62	16,5	
Pouco frequente	73	16,0	92	24,5	
Frequente	296	64,8	222	59,0	
Frutas					< 0,001
Nunca ou raramente	105	23,0	58	15,4	
Pouco frequente	146	32,0	100	26,6	
Frequente	206	45,1	218	58,0	
Leite e queijos					0,019
Nunca ou raramente	130	28,5	82	21,8	
Pouco frequente	103	22,5	116	30,9	
Frequente	224	49,0	178	47,3	
Embutidos					0,635
Nunca ou raramente	252	55,1	200	53,2	
Pouco frequente	116	25,4	103	27,4	
Frequente	89	19,5	73	19,4	
Ultraprocessados					0,007
Nunca ou raramente	163	35,7	143	38,0	
Pouco frequente	142	31,1	144	38,3	
Frequente	152	33,3	89	23,7	
Carnes bovinas					0,010
Nunca ou raramente	208	45,5	138	36,7	
Pouco frequente	174	38,1	158	42,0	
Frequente	75	16,4	80	21,3	
Carnes suínas					0,002
Nunca ou raramente	385	84,4	285	75,8	
Pouco frequente	56	12,3	62	16,5	
Frequente	15	3,3	29	7,7	
Frango					0,008
Nunca ou raramente	41	9,0	31	8,3	
Pouco frequente	132	28,9	74	19,7	
Frequente	284	62,1	271	72,1	
Ovo					0,343
Nunca ou raramente	80	17,5	56	14,9	
Pouco frequente	105	23,0	102	27,1	
Frequente	272	59,5	218	58,0	
Peixe de água doce					0,681
Nunca ou raramente	63	13,8	43	11,4	
Pouco frequente	81	17,7	65	17,3	
Frequente	313	68,5	268	71,3	

* Teste exato de Fisher.

Na Tabela 4, são apresentadas a frequência de consumo de alimentos regionais em ambas as fases hidrológicas analisadas. Destaca-se que o consumo de todos os alimentos regionais analisados foi estatisticamente maior no período da inundação. Os com maior frequência de consumo nos períodos avaliados são tucumã, beiju e farinha de tapioca, e na fase da inundação, o açaí ganha espaço na mesa de quase metade dos amazonenses.

**Tabela 4:** Frequência de consumo de alimentos regionais, nas fases hidrológicas de seca e inundação. Coari, Amazonas, Brasil, 2022.

Variável	Seca (n = 457)		Inundação (n = 376)		Valor de p *
	n	%	n	%	
Milho					0,001
Nunca ou raramente	375	82,1	276	73,4	
Pouco frequente	66	14,4	65	17,3	
Frequente	16	3,5	35	9,3	
Pupunha	253	55,4	239	63,6	< 0,001
Nunca ou raramente	141	30,9	70	18,6	
Pouco frequente	63	13,8	67	17,8	
Frequente	247	54,1	161	42,8	
Pé-de-moleque	160	35,0	124	33,0	< 0,001
Nunca ou raramente	50	10,9	91	24,2	
Pouco frequente	166	36,3	81	21,5	
Frequente	184	40,3	136	36,2	
Beijú de tapioca	107	23,4	159	42,3	< 0,001
Nunca ou raramente	243	53,2	142	37,8	
Pouco frequente	110	24,1	123	32,7	
Frequente	104	22,8	111	29,5	
Farinha de tapioca	425	93,0	265	70,5	< 0,001
Nunca ou raramente	25	5,5	59	15,7	
Pouco frequente	7	1,5	52	13,8	
Frequente	319	69,8	93	24,7	
Uchi	108	23,6	102	27,1	< 0,001
Nunca ou raramente	30	6,6	181	48,1	
Pouco frequente	400	87,5	252	67,0	
Frequente	52	11,4	65	17,3	
Açaí	5	1,1	59	15,7	< 0,001
Nunca ou raramente	389	85,1	279	74,2	
Pouco frequente	59	12,9	53	14,1	
Frequente	9	2,0	44	11,7	
Piquiá	240	52,5	172	45,7	< 0,001
Nunca ou raramente	137	30,0	94	25,0	
Pouco frequente	80	17,5	110	29,3	
Frequente	282	61,7	195	51,9	
Bacaba	122	26,7	99	26,3	< 0,001
Nunca ou raramente	53	11,6	82	21,8	
Pouco frequente	356	77,9	240	63,8	
Frequente	82	17,9	72	19,2	
Tucumã	19	4,2	64	17,0	< 0,001
Nunca ou raramente	385	84,4	285	75,8	
Pouco frequente	56	12,3	62	16,5	
Frequente	15	3,3	29	7,7	
Cuscuz					0,001
Nunca ou raramente	41	9,0	31	8,3	
Pouco frequente	132	28,9	74	19,7	
Frequente	284	62,1	271	72,1	
Carne de caça					< 0,001
Nunca ou raramente	80	17,5	56	14,9	
Pouco frequente	105	23,0	102	27,1	
Frequente	313	68,5	268	71,3	

* Teste exato de Fisher.

## Discussão

Investigações que buscam avaliar o consumo alimentar, as suas transformações por meio do processo de modernização e que incluem aspectos da cultura e da regionalidade são fundamentais, sobretudo no Brasil, cuja diversidade é bastante significativa e deve ser reconhecida e considerada no planejamento e implementação de políticas públicas e estratégias de promoção à saúde.

Diferentemente dos resultados constatados em nível nacional ^17^ ^,^ ^18^, em que o aumento do consumo de ultraprocessados caracteriza a transição alimentar e nutricional, destaca-se o protagonismo dos alimentos *in natura* (frutas, legumes e verduras) nas refeições da amostra deste estudo, em contrapartida a um consumo inferior de alimentos processados e ultraprocessados. Corroborando em parte com nossos achados, um recente inquérito populacional de saúde com indivíduos ribeirinhos da Região Amazônica encontrou baixa frequência ^19^, ou seja, menos de duas vezes por mês, no consumo de alimentos industrializados e refeições prontas entre 67% dos entrevistados. No entanto, os autores ressaltam nesse inquérito que esses produtos que não fazem parte da produção local têm ganhado espaço na região, inclusive em áreas mais remotas.

Tanto a oferta como os preços de alimentos na Região Amazônica são influenciados pela sazonalidade dos rios ^11^, principalmente nos períodos extremos de seca e inundação ^20^. Apesar da existência de transportes fluviais para o deslocamento desses de Manaus (capital do estado) para o interior, os períodos sazonais influenciam na disponibilidade e no acesso de diversos alimentos, como hortaliças, tubérculos e frutas em determinadas localidades da região ^21^.

Neste estudo, ficou evidente a influência da sazonalidade no perfil de consumo da amostra estudada quando se observa consumo maior de alimentos regionais no período da inundação. Uma das possíveis explicações para isso é o fato do lanche da tarde ser realizado por uma proporção maior dos entrevistados nesse período hidrológico (70,2% contra 60,2% no período da seca), considerando os hábitos culturais de consumo desses alimentos regionais. Botelho et al. ^22^ também encontraram um maior consumo de alimentos regionais como peixe, farinha de mandioca e frutas na fase hidrológica da inundação, mas contrariamente, alimentos como peixe e frutas tiveram menor consumo no período da seca.

Portanto, parece que a menor disponibilidade e, consequentemente, o menor consumo de alimentos regionais no período da seca podem estar associados à maior participação dos alimentos ultraprocessados e processados nesse período hidrológico, resultado das comodidades a eles relacionadas como baixo custo e fácil acessibilidade em áreas urbanas ^9^. Na Amazônia, há uma limitação aos diversos recursos devido à localização geográfica e à acessibilidade na região. Se, por um lado, ainda existe a interação entre a sociedade e natureza, refletindo uma dinâmica local própria, por outro, o acesso a bens industrializados (principalmente alimentícios) encurta a distância e as diferenças regionais, homogeneizando os hábitos alimentares ^9^.

O padrão alimentar dos indivíduos exerce grande influência nos parâmetros de saúde da região em que vivem e, apesar do baixo consumo de alimentos ultraprocessados identificado, torna-se relevante a realização de novos estudos voltados à disponibilidade e ao consumo desses na região, com enfoque também nas alterações do clima que têm sido observadas na área e que, consequentemente, influenciam também nos períodos de cheia e vazante e na disponibilidade de alimentos ^23^, sejam aqueles produzidos na região ou os transportados via fluvial de outros locais.

Aprofundar os estudos nesse tempo de transições, de clima e de padrão alimentar é imprescindível para embasar o direcionamento de políticas públicas que fomentem o acesso aos alimentos *in natura*, principalmente os regionais, considerando-se as características de acesso remoto de municípios amazônicos e as evidências sobre os prejuízos à saúde acarretados pelo consumo de alimentos ultraprocessados, que contribuem para o aumento de obesidade, doenças crônicas não transmissíveis e alguns tipos de cânceres ^3^ ^,^ ^17^ ^,^ ^18^.

Como limitação deste estudo, pode-se mencionar o emprego do instrumento TFCA não validado previamente, embora tenha sido utilizado em estudo anterior já publicado ^24^. O TFCA foi selecionado principalmente porque inclui alimentos regionais frequentemente ausentes em outros instrumentos validados. Apesar disso, acredita-se que a utilização dessa estratégia metodológica não invalida os resultados aqui obtidos, importantes e necessários para caracterizar o consumo alimentar de uma população tão pouco estudada em nosso país como a da Região Amazônica.

Outro fator limitante nesta investigação foi a diferença do número de indivíduos entrevistados nos períodos analisados, sendo que o número alcançado foi maior no período de seca quando comparado ao da inundação. Contudo, ressaltamos que isso não inviabilizou os nossos resultados, uma vez que o cálculo amostral foi realizado levando em consideração essas perdas por meio do efeito do desenho *deff* igual a dois e acréscimo de 20% para taxa de não resposta. Na amostra final, tivemos ainda um número superior de indivíduos entrevistados do sexo feminino com relação ao sexo masculino. Nos dias atuais, apesar de as mulheres terem conquistado seu espaço no mercado de trabalho, em regiões remotas e distantes de grandes centros em que as oportunidades de empregos são escassas, como é o caso da Região Amazônica, elas ainda assumem o papel de cuidadoras do lar, o que justifica a predominância do sexo feminino em nossa amostra. Salientamos que essa diferença entre o número de homens e mulheres não invalidou os achados dessa investigação, uma vez que a alimentação é a mesma no contexto familiar, variando apenas em quantidade de pessoa para pessoa. Contudo, recomenda-se para estudos futuros que o desbalanceamento entre o sexo seja considerado no momento da análise estatística.

## Conclusão

Entre os adultos de Coari, município de acesso remoto no Amazonas, predomina o consumo de alimentos *in natura* ou minimamente processados. Há uma importante participação de alimentos regionais no período hidrológico da inundação, em contrapartida a uma participação maior dos alimentos processados e ultraprocessados no período da seca, ainda que pequena em relação aos alimentos *in natura*. Ratifica-se, neste estudo, a influência da sazonalidade dos rios no consumo alimentar da Região Amazônica, dentre elas as fases hidrológicas de seca e inundação.

Pesquisas em saúde que buscam avaliar o consumo alimentar da população no contexto socioeconômico, cultural e regional precisam ser realizadas com mais frequência, de modo a embasar a construção de políticas públicas que considerem as peculiaridades culturais e sazonais nas quais as populações estão inseridas, visando à redução do consumo de alimentos ultraprocessados e dos déficits nutricionais e contribuindo para a prevenção de obesidade e outras doenças crônicas não transmissíveis.
